# Early Postoperative Death in Patients Undergoing Emergency High-Risk Surgery: Towards a Better Understanding of Patients for Whom Surgery May not Be Beneficial

**DOI:** 10.3390/jcm9051288

**Published:** 2020-04-29

**Authors:** Geeta Aggarwal, Katherine J. Broughton, Linda J. Williams, Carol J. Peden, Nial Quiney

**Affiliations:** 1 Royal Surrey County Hospital, Guildford GU2 7XX, UK; nialquiney@nhs.net; 2 Glasgow Royal Infirmary, Glasgow G4 0SF, UK; katherinebroughton@hotmail.com; 3 Usher Institute of Population Health Sciences and Informatics, University of Edinburgh, Edinburgh EH8 9AG, UK; linda.williams@ed.ac.uk; 4 Keck School of Medicine, Los Angeles, CA 90033, USA; carol.peden@med.usc.edu

**Keywords:** postoperative death, emergency general surgery, laparotomy

## Abstract

The timing, causes, and quality of care for patients who die after emergency laparotomy have not been extensively reported. A large database of 13,953 patients undergoing emergency laparotomy, between July 2014 and March 2017, from 28 hospitals in England was studied. Anonymized data was extracted on day of death, patient demographics, operative details, compliance with standards of care, and 30-day and in-patient mortality. Thirty-day mortality was 8.9%, and overall inpatient mortality was 9.8%. Almost 40% of postoperative deaths occurred within three days of surgery, and 70% of these early deaths occurred on the day of surgery or the first postoperative day. Such early deaths could be considered nonbeneficial surgery. Patients who died within three days of surgery had a significantly higher preoperative lactate, American Society of Anesthesiologists Physical Status (ASA-PS) grade, and Physiological and Operative Severity Score for the enumeration of Mortality and morbidity (P-POSSUM). Compliance with perioperative standards of care based on the Emergency Laparotomy Collaborative care bundle was high overall and better for those patients who died within three days of surgery. Multidisciplinary team involvement from intensive care, care of the elderly physicians, and palliative care may help both the communication and the burden of responsibility in deciding on the risk–benefit of operative versus nonoperative approaches to care.

## 1. Introduction

An emergency laparotomy is an urgent major operation that involves an incision of the abdomen to obtain access to the abdominal cavity. Emergency laparotomy is a common operation worldwide, with approximately 30,000 operations undertaken in England and Wales per year [1]. Mortality has improved in the UK in the past few years following national focus and action on this high-risk population and now averages around 9.5% [1,2]. In other countries, mortality is also high and has been reported at rates up to 19% at 30 days [3,4]. These mortality rates are much higher than for similar procedures undertaken electively, where a 30-day mortality of approximately 1–4% is expected [5]. Associated with the high mortality for emergency laparotomy is a wide variation in outcomes related to both patient population [6] and to the quality of care delivered by individual hospitals [7]. Improving standards of care for patients undergoing emergency laparotomy has been successfully addressed in a number of quality improvement projects [8,9] with associated improvement in outcomes. However, little attention has been paid to examining and characterizing patient variability.

The causes of mortality related to patient variation are multifactorial. Patients can often be frail [10] and or elderly and may have significant co-morbidities [11]. Many patients present with advanced diseases such as widespread malignancy [12] or are at high risk of mortality with conditions such as mesenteric ischemia [13]. It is not uncommon for patients to undergo surgery at the end of life, and in the US, 18% of patients over 65 who die undergo surgery within their last month of life [14]. A recent paper exploring end-of life-surgery for nontrauma patients undergoing high-risk emergency general surgical procedures raised the concept of futile surgery [3]. Deciding which surgical patients are most likely to benefit from surgery is difficult [15]. Some major emergency operations may not prolong life but, instead, may increase patient suffering at the end of their life. Patients admitted to the critical care unit near end-of-life report worse quality of life and higher rates of physical distress, whilst not meeting their own personal goals [16]. Considering the patient’s wish to maintain quality of life should be the primary goal for high-value surgical care. Nonbeneficial surgery, where life expectancy is not significantly improved but where patient suffering may be increased, is contrary to this aim [16].

Most publications on emergency general surgery outcomes report in-hospital deaths and deaths at 30 and 90 days. However, less has been described about the timing of death following emergency laparotomy. Some patients will die soon after an emergency laparotomy, whilst others will develop complications and die several weeks later. Modern medicine is skilled at prolonging life, and national standards of care for emergency laparotomy in the UK [1] require all patients with a predicted risk of death greater than 5% to be admitted to an intensive care unit after surgery. Therefore, patients who die in the first few days after emergency surgery are a subset of patients in whom, assuming care is optimal, the physiological stress, underlying comorbidity, the condition requiring surgery, and the impact of the surgery itself are too great to survive. For these patients, the intervention has not been beneficial. For other patients, there may be the potential for recovery, although, for many, development of complications in the days following surgery may lead to death over a longer period. A study of complications after emergency laparotomy found that nearly all patients had complications at day three postoperatively, and the incidence, extent, and type of complication was the same for patients of all ages, but older patients (those over 80) showed markedly decreased survival, suggesting that physiological reserve is key [17]. The aim of this study was to report early deaths after emergency laparotomy and to better understand the characteristics of patients who died in the early postoperative period. We chose the first 72 h as a critical period. A recent study of 94,000 patients who underwent emergency general surgery showed that, of those who died within 31 days, 36% died within 48 h and 45% within 72 h [3]. The rate of death over the subsequent 28 days to 31 days was much slower. In our study, patient factors and care standards of those who died within 72 h were examined and compared with patients who survived the immediate postoperative period.

## 2. Materials and Methods

Ethical approval was sought and was found not to be required for the analysis of the database.

Analysis was carried out on a large database of patients undergoing emergency laparotomy in the 28 hospitals in the South of England that participated in the Emergency Laparotomy Quality Improvement Collaborative (ELC) [2]. Participation in the ELC meant that patients received six evidence-based care bundle components. There was a baseline period before implementation of the care bundle of 15 months, and then data for the 18 months of implementation was collected. The data in this database was collected prospectively by the 28 hospitals and entered into the mandatory National Emergency Laparotomy Audit (NELA) between July 2014 and March 2017. Definitions for inclusion and exclusion to the database were set by NELA [1].

Data entry into the National Emergency Laparotomy Audit [1] requires all patients to have a risk of death calculated prior to surgery using a validated tool such as the Physiological and Operative Severity Score for the enumeration of Mortality and morbidity (P-POSSUM) [18]. P-POSSUM uses physiological parameters and some biochemistry results, as well as age and some estimated operative parameters, to give an indication of risk of death. It is used to guide care, such as admission to the ICU postoperatively.

**Figure 1:**
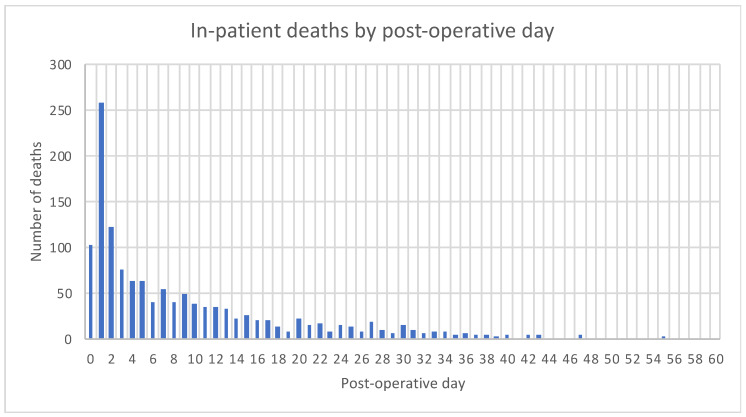
Number of patients that died on days following emergency laparotomy surgery.

**Figure 2:**
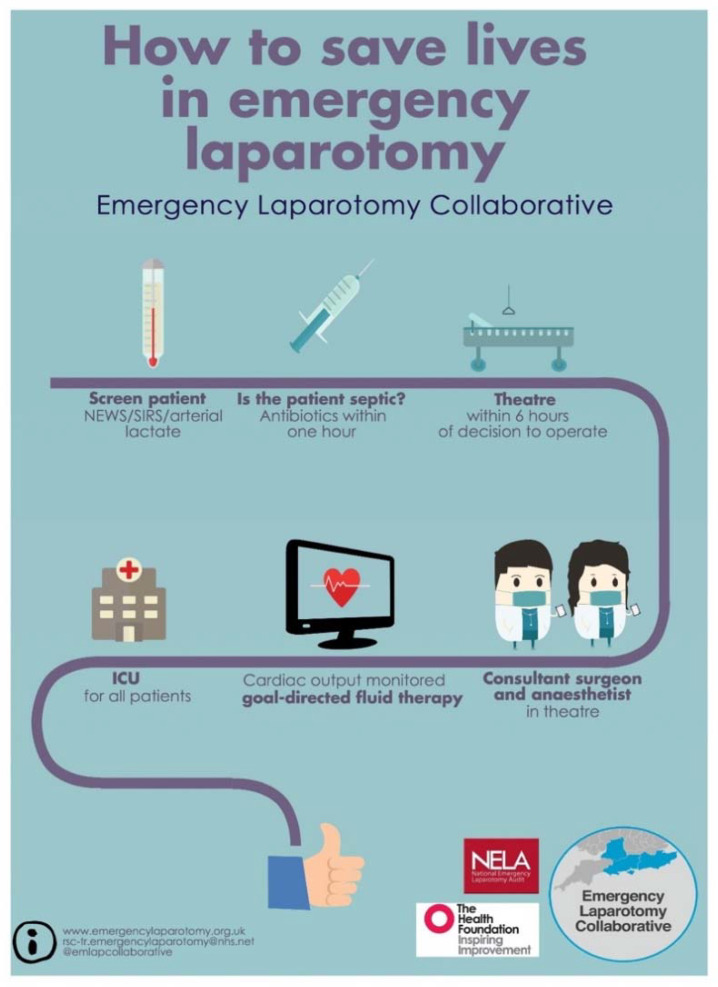
The Emergency Laparotomy Collaborative care bundle implemented in 28 hospitals in the South of England. ICU: intensive care unit, SIRS: systemic inflammatory response syndrome, NEWS: National Early Warning Score.

The time of death (number of whole days between date of surgery and date of death) following surgery was recorded and plotted. A peak of early mortality of patients who died within 3 days of surgery was identified and labeled as “early deaths” (Figure 1). All other patients who survived more than 3 days, labeled “all others”, were then compared with the “early deaths”. In-patient deaths were capped at 60 days. Patient demographics, preoperative physiological observations, preoperative predicted pathology, intraoperative surgical findings, and standards of care were compared between these two groups (Table 1 and Table 2). The standards that were used to assess the quality of care patients received have been used in two other successful quality improvement projects. [2,8] (Figure 2).

### Statistical Analysis

The data was analyzed with SAS (SAS, v9.4, Buckinghamshire, UK) and SPSS (IBM, v22, Portsmouth, UK). The first analysis compared “early deaths” patients with “all others”. A univariate χ^2^ test or Fisher’s exact test (for categorical data) or Kruskall-Wallis test (for continuous data) allowed us to limit the multivariate logistic regression to only the variables that were univariately significant. Forward conditional stepwise regression was used to determine the significant variables, with the probability (*p*-value) for entry = 0.05 and the probability for removal = 0.10. This model was checked using backwards regression to confirm the significant variables, with the same entry and removal probabilities. Multicollinearity was not considered to be an issue, as we only included variables that were independently predictive (and not the entire set of potential predictors). We were less interested in the effects of the individual predictors than in the set of predictors’ ability to predict the outcome as a whole, which is unaffected by multicollinearity.

## 3. Results

### 3.1. In-Patient Mortality 

Thirteen thousand nine hundred and fifty-three patients underwent emergency laparotomy between July 2014 and March 2017. This included 15 months of baseline data (where no intervention of the ELC care bundle took place) and 18 months of intervention data from the ELC care bundle. The overall in-patient mortality was 9.8% (1367/13,953). The 30-day mortality was 8.9% (1242/13,953). Thirty-eight-point one percent of those who died, (519/1363) did so within three days of surgery (“early deaths”). Of the (38.1%) deaths within 3 days of surgery, 70.1% (363/519) occurred on the day of surgery or the first postoperative day. The “early deaths” group was then compared to the all other patients who survived more than 3 days after surgery (“all others”) group.

### 3.2. Demographics and Physiological Variables

Patients who were in the “early deaths” group were significantly older and had a higher American Society of Anesthesiologists Physical Status (ASA-PS) grade when compared to the “all others” group. They also had higher predicted mortality with preoperative P-POSSUM scores [18] with evidence of greater physiological compromise, such as significantly higher arterial lactate, raised serum creatinine, decreased systolic blood pressure, and increased heart rate (Glasgow Coma Score or GCS) (Table 1).

### 3.3. Surgical Indications and Findings

Patients in the “early deaths” group were more likely to have a preoperative diagnosis of a bowel perforation (36.2%), peritonitis (33.3%), or intestinal ischemia (26.4%). The “all others” patient group were more likely to have a preoperative diagnosis of small bowel obstruction (SBO) (35.5%), intestinal obstruction (22.6%), and perforation (22.5%).

The “early deaths” group had a higher frequency of perforation of the small bowel or colon (35.8%) or intestinal ischemia (33.5%). In the “all others” patient group, the most common findings were adhesions (27.3%), perforation of the small bowel or colon (19.9%), and intestinal ischemia (10.9%).

### 3.4. Standards of Care

The standards of preoperative and intraoperative care are shown in Table 2. Overall, high standards of care were delivered to all patients undergoing an emergency laparotomy. Patients in the “early deaths” group were significantly more likely to have an arterial lactate carried out, have both a senior surgeon and anesthesiologist present during surgery, have goal-directed fluid therapy perioperatively, and be admitted to a critical care unit in the postoperative period. There was a higher percentage of patients in the “early deaths” group who received antibiotics at least six hours prior to surgery. The median time to surgery for the group that died within 3 days was shorter at 25 h, compared with 34 h for those that survived longer; however, some patients waited a considerable time for surgery, skewing the data and resulting in a longer mean time to surgery for the early deaths group. In addition, there was a lower compliance in reaching the operating room within two hours (when clinically indicated) in those patients who died within 3 days of surgery.

### 3.5. Univariate and Multivariate Analysis

Variables that were found to have significant differences after univariate analysis were then used in a multivariate analysis. Table 3 shows the factors that were found to have both a positive and negative correlation with “early deaths”. A detailed analysis of this data can be found in Appendix A. Factors that tended to be predictive of “early deaths” were found to be increasing age, raised ASA grade, log of serum creatinine, and surgical findings of bowel perforation or bowel ischemia. Factors that were associated with a decreased risk of “early deaths” following surgery were increased systolic blood pressure, increased Glasgow Coma Score, and log of time to operating theatre.

## 4. Discussion

In this large cohort of patients undergoing emergency laparotomy, almost 40% of the patients who died did so within three days of surgery. This is in line with the findings of other large studies of patients undergoing emergency general surgery [3]. We propose that death early after surgery reflects sustained physiological insult from the underlying condition, which for that individual patient, perhaps due to comorbidity and the time course of the intervention, was not survivable. This group of patients represents 3.7% of the total patient population studied. The term “nonbeneficial” was originally defined as “treatments in those patients who do not survive to hospital discharge” [20]. For patients who died within 72 h of surgery, the procedure could be considered nonbeneficial. Patients who died within three days of an emergency laparotomy had an increased predicted risk of death (increased age, P-POSSUM, lactate, and ASA grade) and were physiologically more compromised (lower systolic blood pressure, raised creatinine, and raised heart rate). There was close correlation between the predicted preoperative surgical diagnosis and intraoperative surgical findings.

In previous publications, poor standards of care were identified as a possible cause of death following emergency laparotomy [4,7]. In the population examined, standards of care for the “early deaths” group were high and significantly better than the “all others” patient group, with over 95% of patients receiving care from a senior surgeon and 92% being admitted to the critical care unit following surgery [2]. One area identified for potential improvement was the time to surgery. The median time to surgery for the group that died within three days was shorter than the survivor group; however, there was a lower compliance in reaching the operating room within two hours (when clinically indicated) in the nonsurvivor patients. This delay is surprising, as this group of patients died from peritonitis, perforation, or ischemic bowel; all three of these conditions are time-critical, and it may be that, if surgery had been sooner, the operation may have been survivable. In addition, there were some patients in the group that survived less than three days with times to theater of over four days. The decision to operate in those patients may have been difficult, resulting in the observed delays. Diagnosis may also have been more difficult. There is clearly a balance that is required between diagnostic investigations, timely surgery, and detailed discussions with the patient and family with regards to risk. Data from the NELA report 2020 [1] indicates that patients who are not admitted under a surgical team may wait up to eight times longer for a consultant surgeon review. We do not have that data for our patients, but presentation to a medical team with a complex history could well be a factor in some cases. The potential to improve outcomes for this very high-risk subgroup may be limited, but for these high-risk patients, time is of the essence. For all clinical teams who come into contact with these patients, the time-critical nature of intervention and management of physiological derangement for patients with an acute abdomen may be one area where further improvement is possible and must continue to be reinforced.

Many patients undergoing emergency laparotomy are elderly and can be frail [21]. Neither the P-POSSUM tool nor the National Emergency Laparotomy Audit risk model [1] included any measure of frailty at the time of this study. Both these models have been assessed against outcomes extensively; however, it is now recognized that frailty is another factor that needs to be considered in outcome prediction. There are many frailty assessments tools available [22], the most widely used being the Clinical Frailty Score developed by Rockwood [23]. All frailty scores can be used to predict the length of stay, postoperative complications, and mortality at 30 days, 60 days, and one year [10]. The measurement of frailty should become part of the routine data collection for all patients undergoing emergency laparotomy. High frailty scores should trigger further consultation with other healthcare professionals and with the patient and relatives in order to improve preoperative decision-making. One approach could be to increase involvement of a multidisciplinary team (MDT) in the preoperative period. MDT discussions of elective surgical oncology patients are now widespread, with studies demonstrating an improvement in diagnostic accuracy and treatment plans [24,25]. There are obvious logistical difficulties to organizing such meetings in an emergency setting. However, involving anesthesiologists, critical care, and elderly care physicians in assessing the risks of surgery should be seen as ideal, and some centers have already achieved this, using triggers to flag high-risk patients [26]. Ideally, the patient should be involved in the decision-making process, and a fully informed discussion should include a discussion about quality of life following discharge. However, although we know that many patients die within a few months of discharge [27], there is only sparse data about their intervening quality of life in terms of pain, nutrition, mobility, and independence [28].

Cooper et al. highlighted some of the reasons for carrying out surgery that may not have benefits, citing surgeon, patient, surrogate, and structural factors [15]. The surgeon may feel everything possible should be done whatever the likelihood of success, especially when the alternative is almost certain death. The surgeon may have limited time for discussion due to the urgency of the case or may feel uncomfortable discussing palliative care [28]. Patients—or, more often, their advocates—may not fully understand what the patient’s best interests might be [15]. Advocates tend not to believe physicians’ predictions and find the concept that ongoing medical treatment would be futile difficult and may wish to continue treatment despite no chance of survival being predicted [29,30]. These factors can lead to nonbeneficial surgery and poorer end-of-life care. Leaving the physician as the ultimate arbiter of futility is neither helpful nor appropriate. The principle of patient autonomy recognizes the right to self-determination, and where predicted risks of death or substantial morbidity are high, the patient should be encouraged to identify “what matters to them” [31]. At present, a binary approach is often offered to patients in terms of death or survival. However, survival will, in all probability, be complicated for these high-risk patients, with lengthy critical care and hospital stays and impaired long-term quality of life [28]. The concept of what conditions patients perceive are “worse than death” is a relatively new one [32], but it has important messages for clinicians involved in making difficult decisions with patients about to undergo high-risk emergency surgery. Several initiatives have been launched to help clinicians develop skills to actively involve patients’ wishes and undergo difficult end-of-life conversations. The use of “shared decision-making tools” have been shown to be associated with improved patient knowledge of outcomes [31,33].

There are a few limitations to this study. Death within three days was used as a marker for nonbeneficial surgery. However, if we use the original definition of patients not surviving to hospital discharge [20], there will be other patients who did not benefit from surgery. The information within the National Emergency Laparotomy dataset [1] at the time of this study did not include questions on frailty scores, admitting specialty, and the treatment of conditions such as sepsis; these have now been added. The causes of death in the “early deaths” group is not known, and data is not captured on patients with acute abdomens who fulfilled the NELA criteria but did not undergo surgery. There has been little study of patients who present with acute abdomen but do not undergo an emergency laparotomy. A recent paper from a single center showed that the mortality rate in patients who would qualify for an emergency laparotomy under the NELA criteria but did not undergo surgery was 63% at 30 days, which was higher than the mortality risk generated using a risk-score model, suggesting that some patients may have benefitted from surgery [34].

Despite the improvement in standards of care across the UK for patients undergoing emergency laparotomy, mortality is still high. We believe that attention should be turned to identifying patients where surgery may not be of benefit. Better methods, such as the routine assessment of frailty, to identify patients most at risk of early death after surgery need to become routine. Decisions about operating or not are very hard, and support and coaching for improved decision-making needs to be developed. The use of multidisciplinary teams with individuals skilled in better understanding the expectations and wishes of individual patients should be promoted. Finally, the reporting of death within three days of surgery should become standard, together with mortality at 30 and 90 days. This would allow clinicians to focus on patient selection and promote more holistic end-of-life care.

## 5. Conclusions

This study is one of only a small number of articles investigating the timing of death in patients who have undergone emergency laparotomy. Thirteen thousand nine hundred and fifty-three patients were analyzed in the database, and an in-patient mortality rate of 9.8% was found. Thirty-eight-point one percent of patients who died did so within three days of emergency surgery, and of those, 70.1% died within one day of surgery. Analysis of these patients showed an older age, a higher P-POSSUM, lactate, and ASA grade, and they were more physiologically compromised, with a lower systolic blood pressure, raised creatinine, and raised heart rate. Usual risk-prediction tools are poor at predicting early deaths from surgery, and communication and support for surgeons regarding this emotive issue is needed. Multidisciplinary team involvement from intensive care, care of the elderly physicians, and palliative care may help both the communication and the burden of responsibility in deciding on the risk–benefit of operative versus nonoperative approaches to care. 

## Figures and Tables

**Table 1:** Characteristics of patients who either died within 3 days (early deaths) or all others.

	“All Others” (*n* = 13,434)	“Early Deaths” (*n* =519)	*p*-Value
Mean Age (IQR)	68 (54–78)	75 (67–83)	<0.0001
Gender			
Male (%)	6284 (47%)	250 (48%)	0.53
Female (%)	7150 (53%)	269 (52%)	
Lactate (IQR)	1.3 (1.0–2.1)	3.6 (1.7–7.1)	<0.0001
Pre-op P-POSSUM mortality (range)	6.2 (3.5–18.9)	54.2 (23.4–80.6)	<0.0001
Post-op P-POSSUM mortality (range)	6.5 (2.5–18.9)	53.5 (27.1–80.8)	<0.0001
ASA-PS (range)	3 (2–3)	4 (3–4)	<0.0001
Creatinine (range)	75 (60–97)	115 (81–165)	<0.0001
Sodium (range)	137 (134–139)	136 (132–140)	0.01
WCC (range)	11.0 (7.8–15.2)	11.7 (6.8–18.0)	0.15
Systolic BP (range)	127 (112–140)	110 (90–130)	<0.0001
Heart rate	88 (77–100)	100 (88–120)	<0.0001
GCS (range)	15 (15–15)	15 (14–15)	<0.0001
Potassium (range)	4.1 (3.8–4.5)	4.2 (3.8–4.8)	<0.0001

BP: blood pressure; GCS: Glasgow Coma Score; P-POSSUM: Physiological and Operative Severity Score for the enumeration of Mortality and morbidity; ASA-PS: American Society of Anesthesiologists Physical Status; WCC: white cell count.

**Table 2:** Compliance of patients with the standards of care outlined by the Emergency Laparotomy Collaborative care bundle for patients undergoing emergency laparotomy.

	“Early Deaths”	“All Others”	*p*-Value
Number	519	13,434	
Preoperative lactate measured	89.2%	69.9%	<0.0001
Time from admission to surgery (median) Time from admission to surgery (mean)	25 h – – – – 4.5 days	34 h – – – – 3.4 days	0.07 – – – – <0.0001
Antibiotic therapy administered at least 6 h prior to surgery [2]	32.1%	28.4%	0.06
Preoperative CT scan performed	83.4%	86.1%	0.09
Met NCEPOD-based target of surgery <2 h [19]	44.1%	55.0%	<0.0001
Intraoperative goal-directed fluid therapy	70.3%	64.0%	0.003
Postoperative critical care admission	90.8%	88.6%	<0.0001
Consultant/attending surgeon present for operation	93.3%	88.6%	0.001
Consultant/attending anesthesiologist present for operation	90.8%	81.9%	<0.0001

NCEPOD: National Confidential Enquiry into Patient Outcome and Death.

**Table 3:** Characteristics that compose positive and negative predictors of early deaths in emergency laparotomy. A full analysis can be found in Appendix A.

Positive Predictors of “Early” Deaths	Negative Predictors of “Early” Deaths
Age	Systolic blood pressure
Log of postoperative P-POSSUM	Glasgow Coma Score
ASA-PS score	Log of time to theater
Log of creatinine	
A surgical finding of intestinal ischemia	
A surgical finding of perforation of the small bowel	

P-POSSUM: Physiological and Operative Severity Score for the enumeration of Mortality and morbidity; ASA-PS: American Society of Anesthesiologists Physical Status.

## Appendix A

**Table A1:** Multivariate analysis (following stepwise regression).

				95% CI for OR	
*n* = 13,688 (98% of Cases)	df	*p*-Value	OR	Lower	Upper
Age on Arrival	1	0.000	1.022	1.014	1.031
ln_postoperative P-POSSUM	1	0.000	2.173	1.885	2.505
ASA Score (ref = 1)	4	0.000			
ASA Score 2	1	0.726	1.243	0.368	4.195
ASA Score 3	1	0.068	2.967	0.922	9.547
ASA Score 4	1	0.002	6.246	1.936	20.145
ASA Score 5	1	0.000	16.254	4.827	54.734
ln_creatinine	1	0.011	1.274	1.058	1.535
Systolic blood pressure	1	0.000	0.992	0.988	0.997
Glasgow coma score	1	0.001	0.942	0.910	0.975
ln time to theater	1	0.002	0.890	0.827	0.957
Histology (reference = Other)	7	0.000			
Histology (Crohn’s Disease)	1	0.706	0.786	0.224	2.758
Histology (Diverticulitis)	1	0.074	0.629	0.378	1.047
Histology (Ischemia)	1	0.125	0.729	0.487	1.092
Histology (Malignancy)	1	0.698	1.075	0.747	1.546
Histology (PUD)	1	0.086	0.500	0.227	1.102
Histology (UC)	1	0.260	0.309	0.040	2.388
Histology (NA)	1	0.000	0.547	0.397	0.752
Operative findings; Intestinal ischemia	1	0.029	1.451	1.039	2.026
Operative findings; Perforated Small Bowel	1	0.007	1.389	1.093	1.765
Indication for surgery; Ischemia	1	0.000	2.003	1.418	2.829

df: degrees of freedom; *p*-value: probability value; OR: Odds ratio; PUD: peptic ulcer disease; UC: ulcerative colitis; NA: not available.
